# Antimetastatic Effect of Fucoidan-Sargassum against Liver Cancer Cell Invadopodia Formation via Targeting Integrin αVβ3 and Mediating αVβ3/Src/E2F1 Signaling

**DOI:** 10.7150/jca.26740

**Published:** 2019-08-27

**Authors:** Ting-Jia Pan, Li-Xin Li, Jia-Wei Zhang, Zhao-Shuo Yang, Dong-Min Shi, Yun-Ke Yang, Wei-Zhong Wu

**Affiliations:** 1 Department of Traditional Chinese Medicine, Zhongshan Hospital, Fudan University, 180 Fenglin Rd, Shanghai 200032, China.; 2 Liver Cancer Institute, Zhongshan Hospital, Fudan University, Key Laboratory of Carcinogenesis and Cancer Invasion, Ministry of Education, Shanghai 200032, China

**Keywords:** invadopodia, metastasis, liver cancer, fucoidan, integrin, antineoplastic

## Abstract

**Background**: Fucoidan is a fucose-enriched, sulfated polysaccharide found in brown algae; in recent years, this polysaccharide has been found to exert several biological effects, including antitumor effects, such as antiproliferation, activating apoptosis, and anti-angiogenesis of cancer cells. However, the antimetastatic effect of fucoidan and the related targeting receptors remain unknown. In the present study, we examined the inhibition of invadopodia formation and underlying mechanism of fucoidan on human liver cancer cells.

**Methods**: We used 98% purified fucoidan from Sargassum species to treat the hepatocellular carcinoma (HCC) cells SMMC-7721, Huh7 and HCCLM3 *in vitro* and the HCCLM3 cell line *in vivo*. The HCC cells were cultured with various concentrations of Fucoidan-Sargassum (0-30 mg/mL). Migration, invasion and wound healing assays were performed to determine the antimetastatic effect of fucoidan on the HCC cells. Western blot analysis and immunofluorescence staining were conducted to determine the expression levels of invadopodia formation-regulating proteins and the targeting membrane receptor proteins.

**Results**: Fucoidan-Sargassum inhibited the migration and invasion of HCC SMMC-7721, Huh7 and HCCLM3 cells in a dose-dependent manner. In the HCCLM3 cells, Fucoidan-Sargassum also decreased the expression levels of invadopodia-related proteins including Src, Cortactin, N-WASP, ARP3, CDC42, MMP2, MT1-MMP, and the targeting receptors integrin αV and β3 in a dose-dependent manner. Fucoidan-Sargassum also increased the levels of endoplasmic reticulum-related proteins, including GRP78, IRE1, SPARC, and the type IV collagen receptor proteins integrin α1 and β1. *In vivo*, Fucoidan-Sargassum reduced the size of liver tumors and decreased the number of lung metastatic foci in nude mice with hepatocellular carcinoma xenografts.

**Conclusion**: These findings indicate that Fucoidan-Sargassum has an antimetastatic effect on SMMC-7721, Huh7 and HCCLM3 liver cancer cells, and the underlying mechanism involves targeting ITGαVβ3 and mediating the ITGαVβ3/SRC/E2F1 signaling pathway. These results suggest that Fucoidan-Sargassum may be a promising therapeutic antimetastatic compound in the development of a metastasis-preventive drug for treating liver cancer.

## Introduction

Migration is an inherent ability of malignant cancer cells, enabling them to metastasize from a primary site to a secondary site in a scale from uncontrollable proliferation, extracellular matrix (ECM) degradation, subendothelial basement membrane invasion, to distant organ colonization within a host body. During metastasis, cancer cells reprogram gene expression to activate a series of events that are required to complete the process of epithelial-mesenchymal transition (EMT) to mesenchymal-epithelial (re)transition (MET) 1. One of the prominent characteristics of metastatic cancer cells is their ability to invade. Hence, invadopodia formation is an important process for cancer cells to gain migratory and invasive abilities to penetrate through barriers and then to escape into the bloodstream 2. Furthermore, metastasis is positively associated with mortality and is responsible for more than 90% of cancer-related death 3; therefore, metastasis has always been a cause for fear among cancer patients.

Hepatocellular carcinoma (HCC) is one of the common aggressive cancer types; although the morbidity of HCC is ranked the fifth in the world, its mortality is ranked second in cancer-related deaths worldwide 4. The primary reason for the high mortality of HCC is the high rate of late diagnosis after the window for surgical resection has long been closed; at that point, only few options are available to combat the advanced-stage disease. In this context, developing antitumor drugs that possess antineoplastic activity to prevent HCC progression and can be conveniently used in drug therapy is an imperative mission for researchers.

In recent years, natural antitumor compounds have gained increasing attention from patients and researchers for their biological activities, characteristics, and minimal or no side effects. Fucoidan, which is a fucose-enriched sulfated polysaccharide, is isolated from the extracellular matrix of various species of brown algae and primarily consists of L-fucose and sulfate along with small quantities of D-mannose, D-galactose, D-xylose, and uranic acid 5,6,7; the structure varies among different brown algae species. Accumulating evidence indicates the potential biological functions of fucoidan, including antitumor 8, anti-angiogenesis 9, anticoagulant 10, anti-inflammatory 11, and antioxidant effects 12. In cancer-related research, most studies have shown that fucoidan inhibits cancer cell proliferation and induces apoptosis 13. However, most of the research has been focused on the bioactivity of fucoidan isolated from Laminaria (one of the species of brown algae) and its effects against colorectal cancer (clinical phase) 14, lung cancer 15, and breast cancer 16. Whether fucoidan from Sargassum species can suppress metastatic behavior in liver cancer remains unknown. The purpose of this study was to determine the antineoplastic activity of Fucoidan-Sargassum in HCC cell lines and to investigate the possible mechanism underlying the antineoplastic property of fucoidan, which inhibits the migratory and invasive abilities of cancer cells. For this purpose, we selected the HCC cell lines SMMC-7721, Huh7, and HCCLM3 to conduct functional experiments *in vitro*; in addition, we used a hepatocellular carcinoma xenograft model in 5-week-old nude mice to examine the antineoplastic activity of fucoidan *in vivo* through oral administration.

## Materials and Methods

### Reagents and antibodies

98% purity sulfated Fucoidan-Sargassum fusiforme was purchased from Shaanxi Kang Yue Biological Technology co., LTD, and dissolved in Dulbecco's Modified Eagles Medium from Hyclone (Logan, UT, USA) for *in vitro* and PBS for *in vivo* study. Fetal bovine serum was from Gibco (Grand Island, NY, USA). Matrigel matrix was purchased from Corning (Corning, NY, USA). Crystal Violet Staining Solution, Cell Counting Kit-8 were from Beyotime Biotechnology (Shanghai, China). The following mouse monoclonal antibodies were used: anti-cortactin from Millipore (Molsheim, France), anti-GAPDH from Beyotime Biotechnology (Shanghai, China), anti-VCAM1, anti-CD44 from Proteintech (Rosemont, IL, USA). Rabbit polyclonal antibodies were used: anti-Tublin, anti-IRE1a were from Beyotime Biotechnology (Shanghai, China), anti-TKS5, anti-ITGα1 were from Zen-Biotechnology (Sichuan, China), anti-GRP78, aniti-GRP94, anti-SPARC, anti-c-Src were from Proteintech (Rosemont, IL, USA), anti-ITGαV, anti-ITGβ3, anti-ITGβ1, anti-Src, anti-phospho-Src416, anti-phospho-cortactin, anti-FAK, anti-phospho-FAK, anti-ARP2, anti-ARP3, anti-N-WASP, anti-WAVE-2, anti-Rac1/Cdc42, anti-phospho-Rac1/Cdc42(ser71), anti-E2F1, anti-phospho -E2F1, anti-phospho-MPZL1 were from Cell Signaling Technology (Danvers, MA, USA), anti-MPZL was from Abcam (Cambridge, MA, USA). Alexa Fluor 647-conjugated goat-anti-mouse and TRITC-Phalloidin were from Yeasen (Shanghai, China), FITC goat anti-rabbit was from Proteintech (Rosemont, IL, USA), DAPI and Alexa Fluor 647-conjugated goat-anti-rabbit were from Beyotime Biotechnology (Shanghai, China). Anti-Cortactin conjugated 488 was purchased from Abcam (Cambridge, MA, USA).

### Cell lines and culture

Human hepatocellular carcinoma cells Huh7 and SMMC-7721 were gifted from Second Military Medical University, Shanghai, China, and HCCLM3 cell line was established in our laboratory 17; they were cultured in DMEM, supplemented with 10% FBS and antimicrobial (1mL/500mL Primocin, Invivogen, CA, USA). All cells were cultured in 37°C, 5% CO2 humidified incubator.

### Cell viability assay

Exponentially growing HCC cell lines Huh7, SMMC-7721, and HCCLM3 in 96-well plates (5,000 cells/well) were sub-confluently incubated with Fucoidan-Sargassum (0, 10, 20, 30, 40 mg/mL) for various time frames (24, 48, 72 h). Each day, cell viability was determined using Cell Counting Kit-8, a mixture of 10μL CCK-8 solution and 100μL of DMEM (no FBS) was added to each well and incubated for 2h in 37°C, 5% CO2 humidified incubator. Afterward, the optical density (OD) of each well at 450/620 nm was measured using a microplate reader (Molecular Devices, Sunnyvale, CA, USA).

### Wound healing assay

The HCC cell lines Huh7, SMMC-7721, and HCCLM3 were seeded in 6-well plates and grown to 80% confluence in 2mL of growth medium. A 10μL sterile pipette tip was used to scratch a cross mark on the cell monolayer. The cells were subsequently treated with Fucoidan-Sargassum (0, 5, 10, 20mg/mL for Huh7 and 0, 10, 20, 30mg/mL for SMMC-7721 and HCCLM3), then wound closures were observed at 0, 24 and 48 h under an inverted microscope (Olympus, Tokyo, Japan). Four random fields were selected and measured. The migration index was calculated by the ratio of migrating area of treated cells to their counterparts.

### Migration and invasion assays

24-well, 8-μm-pore size Transwell plate (Costar, Cambridge, MA, USA) was used to perform both migration and invasion assays. For migration assay, SMMC-7721, Huh-7 cells (5 × 104 cells/well) and HCCLM3 cells (8 × 104 cells/well) in 100μL of serum-free medium, then added in another 100μL of different dosage of Fucoidan-Sargassum (total 200 μL) seeding in the upper chamber. For the lower chamber, added in 300μL of DMEM with 10% FBS for SMMC-7721 and Huh7 cells, 15% FBS for HCCLM3 cells. After 48 h incubation, the migrated cells were stained with crystal violet, then used cotton swab gently to remove non-migrated cells on the upper surface of the chamber. The digital photos of migrated cells were taken under an inverted microscope (Olympus, Tokyo, Japan). For invasion assay, Matrigel was mixed with 5mg/mL in serum-free cold medium and added 80μL of the mixed solution into each upper chamber, and let it sit in the room temperature for an hour to get harden. Next, seeded cells, SMMC-7721, Huh7 cells (7 × 104 cells/well) and HCCLM3 cells (1.5 ×105 cells/well), then the remaining steps were the same as migration assay, which is described in above.

### Immunofluorescence staining

Cells were seeded at 3000 cells/cm2 in confocal culture plates and incubated overnight at 37°C with 5% CO_2_. Cultured medium was removed, then added DMEM without FBS for control and Fucoidan-Sargassum solution for treatment, then incubated overnight. Cells were first gently washed with PBS and fixed it using 4% paraformaldehyde solution for 10 min, then washed with PBS for 2 min, permeabilized by Saponin for 8 min, washed with PBS for 3 times 5 min each. Unspecific sites were blocked with 5% goat serum in TBS for 30 min at room temperature. Cells were then labeled for overnight at 4°C with, anti-c-Src (1:100), anti-ITGαV (1:100), anti-SPARC (1:50), anti-GRP94 (1:50), and/or with Alexa-488-conjugated cortactin (1:100), and covered in the dark. After three washes with PBS-T, cells were incubated with FITC-conjugated goat anti-rabbit (1:100), Alexa-647-conjugated goat anti-mouse and Alexa-647-conjugated goat anti-rabbit (1:1000) for 1 h, washed three times with PBS-T. Next, labeled with TRITC-Phalloidin (1:300) for 30min at room temperature, washed three times with PBS-T. Afterward, labeled with DAPI for 3 mins, washed 3 times with PBS-T. Lastly, added antifade mounting medium, the images were taken using Leica Microsystems Confocal Microscope (Wentzler, Germany).

### Western blot

Cells treated with four different dosages of Fucoidan-Sargassum in six-well plates were washed with an equal volume of PBS and lysed with RIPA buffer containing 1mM PMSF on the ice and then centrifuged for 15 min at 12000rpm at 4°C. Protein concentration was determined using a Pierce BCA protein assay kit (Waltham, MA, USA) based on the Lowry colorimetric method. Then, 10 μg of protein were suspended in loading buffer, and loaded on a 6%, 8%, and 10% polyacrylamide gel (depending on the targeted protein's molecular weight). After transferred it onto a polyvinylidene difluoride (PVDF) membrane, nonspecific sites were blocked for 2 h at room temperature using 5% non-fat dried milk powder or BSA (for Phosphorylated protein) in TBS-T. After blocking, the membranes were incubated with the antibodies against E2F1, p-E2F1, ARP2, ARP3, N-WASP, ITGα1, ITGβ1, ITGαV, ITGβ3, GRP78, IRE1, SPARC, GAPDH, β-actin, Tublin, Tks5, cortactin, p-cortactin, Src, p-Src family, E-cadherin and Caveolin-1 (diluted at 1:1000 ratio) over night at 4°C. The next day, washing membranes three times in TBS-T for 15 min, then incubated with specific horseradish peroxidase-labeled goat anti-rabbit or anti-mouse secondary antibody (Cell Signal Technology, Danvers, MA, USA) (diluted at 1:5000 ratio) for 1 h at room temperature, and then detected with ECL Detection System (Thermo Scientific, Rockford, IL, USA).

### Cell transfections

Short hairpin RNA (shRNA) against ITGαV, ITGβ3 plasmid, and corresponding negative controls were purchased from OBiO Technology (OBiO, Shanghai, China). These oligonucleotides and vectors were transfected into HCCLM3 cells using Lipofectamine 2000 (Thermo Fisher Scientific, Waltham, MA, USA).

### Animal model

Five-week-old male BALB/c nude mice purchased from Shanghai Laboratory Animal Co. Ltd. (Shanghai, China) were used for implementing hepatocellular carcinoma xenograft model 18. All nude mice underwent a surgical procedure, which embedded a piece of HCCLM3 tumor tissue under fibrous capsule of the liver. After resting for 2 days, the Fucoidan-Sargassum group was orally administrated with 1g/Kg of Fucoidan-Sargassum reagent for 21days, and the control group was orally administrated with PBS. Afterward, mice were sacrificed and liver tumors were harvested, photographed, measured and examined by immunohistochemical staining. Tumor sizes were calculated using the formula: Volume (mm3) = [π × width (mm) × length (mm) × height (mm)]/6^19^. The total metastatic foci were examined by pathological identification. All animal received humane care according to the criteria outlined in the “Guide for the Care and Use of Laboratory Animals” prepared by the National Academy of Sciences and published by the National Institutes of Health (NIH publication 86-23 revised 1985).

### Statistical Analysis

All data were analyzed using GraphPad Prism7 (La Jolla, CA, USA). The Student's t-test was used to analyze differences between two groups, and one-way or two-way ANOVA was used when more than two groups were compared. A two-tailed value of P < 0.05 was considered significant.

## Results

### Fucoidan-Sargassum inhibits the migration and invasion of SMMC-7721, Huh7, and HCCLM3 cells

**Figure 1:**
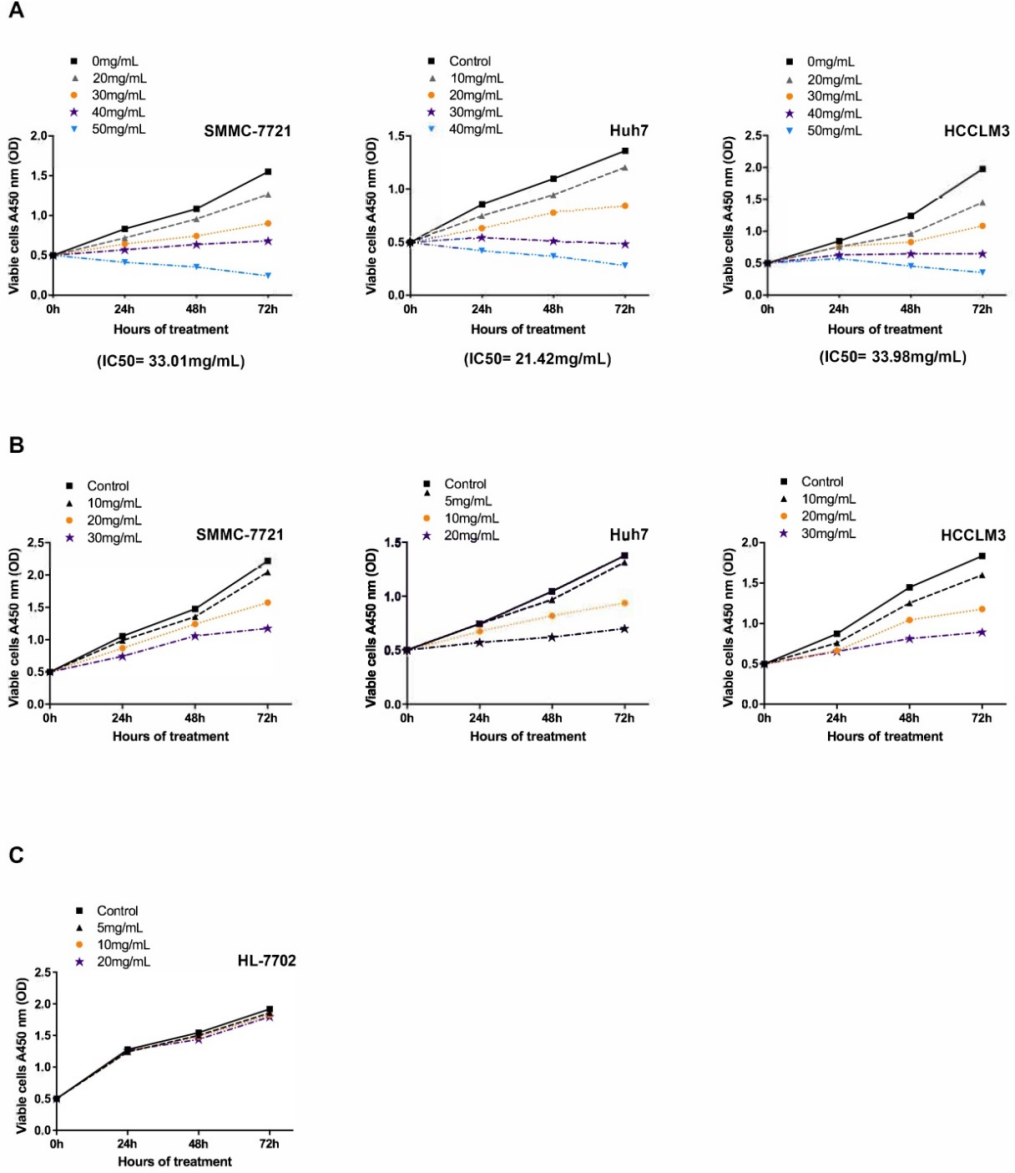
Fucoidan-Sargassum inhibits the proliferation of HCC cell lines SMMC-7721, Huh7 and HCCLM3 but not the normal liver cell line HL-7702. (A) The IC_50_ was determined for the SMMC-7721, Huh7 and HCCLM3 cell lines using the cell viability assay after the cells were treated with the indicated concentrations of Fucoidan-Sargassum for 0 to 72 h. (B) SMMC-7721, Huh7, and HCCLM3 cells were treated for 0-72 h with 4 concentrations of Fucoidan-Sargassum that were under the IC_50_ to examine the dose dependency and inhibition rate (refer to Table 1 for detail statistical information). (C) The normal liver cell line HL-7702 was tested for cytotoxicity with indicated concentrations of Fucoidan-Sargassum (no statistical significance).

To investigate the antineoplastic effect of Fucoidan-Sargassum on the HCC cell lines SMMC-7721, Huh7, and HCCLM3 under viable condition, we performed a cell viability assay to determine the half maximal inhibitory concentration (IC50) of Fucoidan-Sargassum in each cell line. The results revealed that the IC_50_ of Fucoidan-Sargassum in SMMC7721, Huh7, and HCCLM3 cells was 33.01 mg/mL, 21.42 mg/mL, 33.98 mg/mL, respectively (Figure 1A). This finding indicates that Huh7 is slightly more sensitive to Fucoidan-Sargassum compared with the other two HCC cell lines. Next, to study the inhibition rate of Fucoidan-Sargassum according to dose increments and time duration, we prepared four gradient concentrations tailored to each individual HCC cell line based on the IC_50_. The doses for the Huh7 cell line were 0, 5, 10, and 20 mg/mL; and the doses for both the SMMC-7721 and HCCLM3 cells were 0, 10, 20, and 30 mg/mL (Figure 1B). In addition to treating the HCC cell lines, we treated the normal liver cell line HL-7702 to investigate the potential cytotoxicity of Fucoidan-Sargassum. The results showed that Fucoidan-Sargassum inhibits HCC cells in a dose-dependent manner, except in the normal liver cell line HL-7702, and an adequate time duration is 48 h (Table 1).

Next, we used these modified concentrations to study the antineoplastic activity of Fucoidan-Sargassum in a migration assay, invasion assay and wound healing assay.

**Figure 2:**
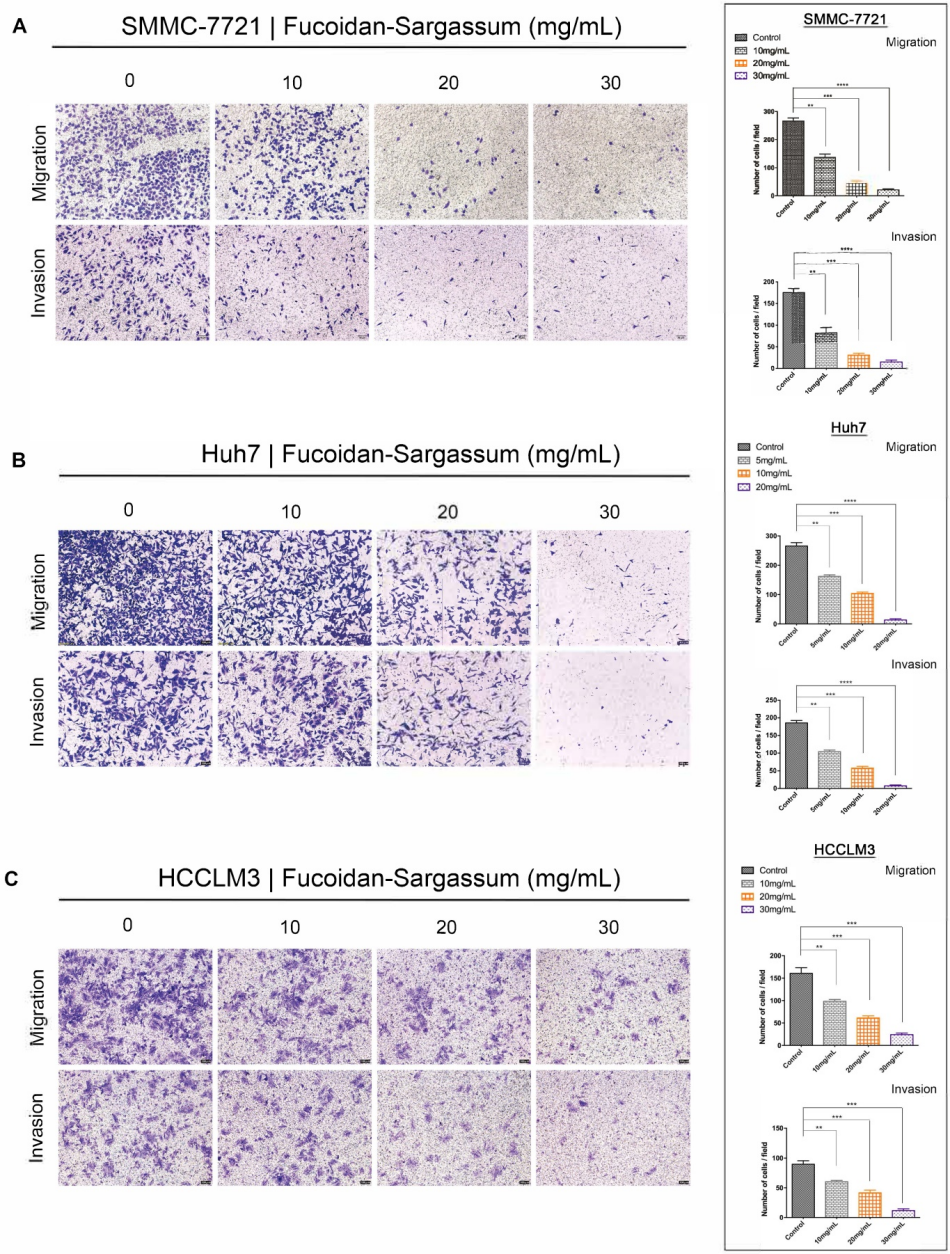

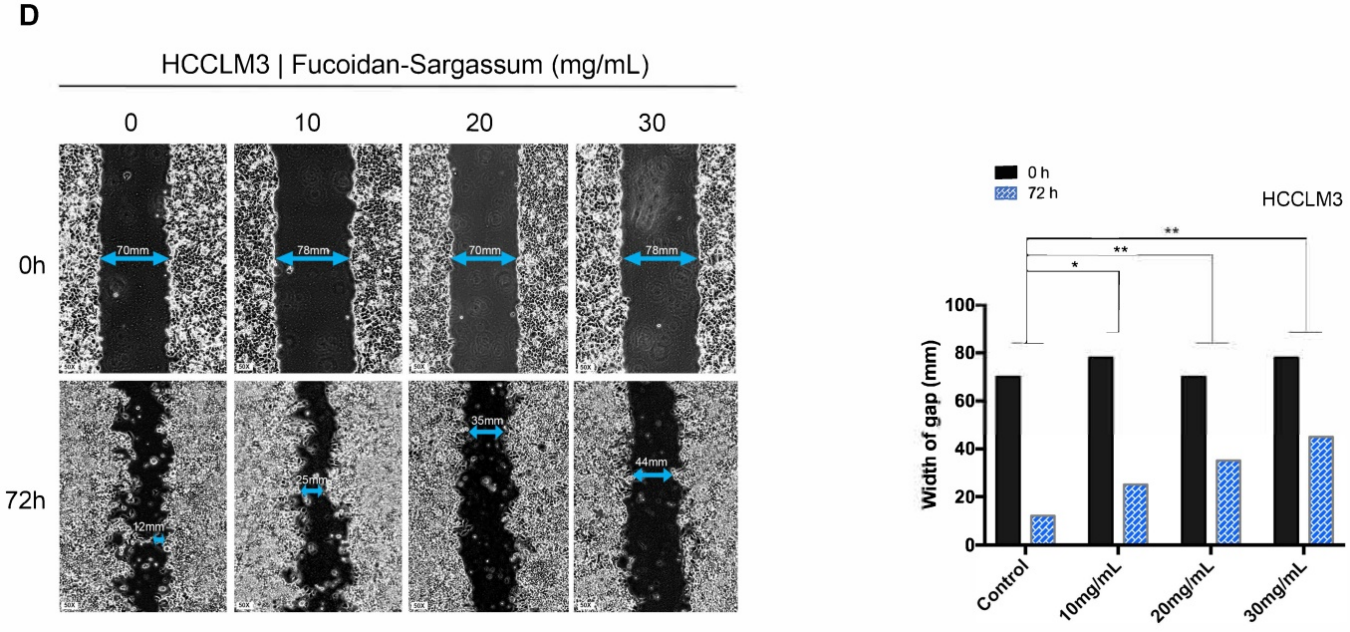
Fucoidan-Sargassum inhibits migration and invasion of HCC, SMMC-7721, Huh7 and HCCLM3 cells in a dose-dependent manner. (A-C) A Transwell assay was performed to evaluate the impact of Fucoidan-Sargassum on the migratory and invasive capabilities of SMMC-7721, Huh7, and HCCLM3 cells. Before seeding the cells into the inner chamber, both SMMC-7721 and HCCLM3 cells were treated with Fucoidan-Sargassum (0, 10, 20, and 30 mg/mL), and Huh7 cells were treated with (0, 5, 10, and 20 mg/mL). After 48 h of incubation in the inner chamber, the migrated and invasive cells were photographed. Each HCC cell line is represented by two bar graphs showing the effectiveness of Fucoidan-Sargassum treatment on inhibiting the migration and invasion of the cells in a dose-dependent manner (*P < .05, **P < .01, ***P < .001, ****P < .0001 vs. control, by ANOVA). (D) The HCCLM3 cells were seeded onto six-well culture plates and grown to 90% confluence in culture medium containing 10% fetal bovine serum. The cells were scratched with a 10 µL pipette tip and subsequently treated with Fucoidan-Sargassum (0, 10, 20, and 30 mg/mL). The images are representative of the inhibitory effect of dose increments of Fucoidan-Sargassum on the migration of HCCLM3 cells (highly metastatic) (top lane; incubation for 0 h, bottom lane; incubation for 72 h) (*P < .05, **P < .01 vs. control, by ANOVA).

Through the migration and invasion assays, we observed that the migratory and invasive abilities of all HCC cell lines were inhibited in a dose-dependent manner. In the migration assay, the migration of the treated SMMC-7721 cells decreased by more than 1.86-, 5.81-, and 12.42-fold, respectively, compared with the control cells (P< .01, P< .001, P< .0001 Figure 2A). In the invasion assay, the invasive capability of the treated cells decreased by more than 2.15-, 5.65-, and 11.67-fold, respectively, compared with the control cells (P < .01, P < .001, P < .0001 Figure 2A). For Huh7 cells, the migration of the treated cells decreased by more than 1.63-, 2.54-, and 19.38-fold, respectively, compared with the control cells (P < .01, P < .001, P < .0001 Figure 2B), and the invasive capability of the treated cells decreased by more than 1.78-, 3.23-, and 24.22-fold, respectively, compared with the control cells (P < .01, P < .001, P < .0001 Figure 2B). Finally, for HCCLM3 cells, the migration of the treated cells decreased by more than 1.63-, 2.62-, 3.80-fold, respectively, compared with the control cells (P < .01, P < .001, P < .0001 Figure 2C), and the invasive capability of the treated cells decreased by more than 1.48-, 2.15-, and 4.56-fold, respectively, compared with the control cells (P < .01, P < .001, P < .001 Figure 2C). Compared to SMMC-7721 and Huh7 cells, HCCLM3 is a more metastatic HCC cell line; thus, we only used HCCLM3 cells in the wound healing assay to further investigate the antineoplastic activity of Fucoidan-Sargassum. In this assay, the untreated cells closed the gap after 72 h, whereas the treated cells never closed the gap, and the width of the gap widened as the concentration of Fucoidan- Sargassum increased (Figure 2D). Based upon these findings, we concluded that Fucoidan-Sargassum inhibits the migratory and invasive abilities of various HCC cell lines in a dose-dependent manner.

### Fucoidan-Sargassum inhibits the initiation, assembly, and maturation stages of invadopodia formation in HCCLM3 cells

**Figure 3:**
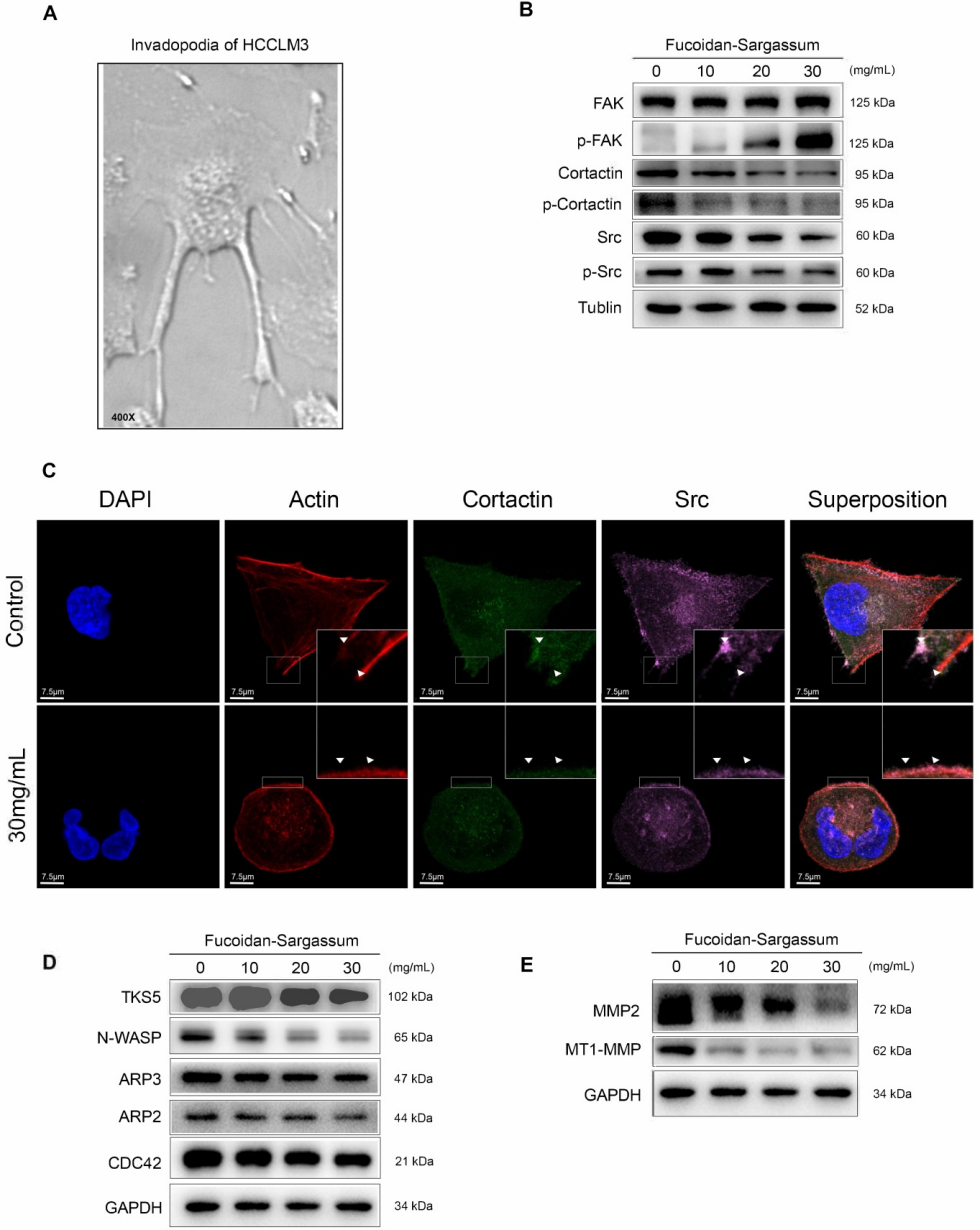
Fucoidan-Sargassum inhibits the initiation and assembly stages of invadopodia formation in HCCLM3 cells. (A) The elongated protrusions of the HCCLM3 cells, which are called invadopodia; the image was taken under an inverted microscope (400× magnification). (B) Western blotting analysis of FAK, Src, and Cortactin, which are key proteins involved in the initiation stage of invadopodia formation, after the cells were treated with Fucoidan-Sargassum (0, 10, 20, and 30 mg/mL) for 48 h. (C) Confocal images of fixed control or Fucoidan-Sargassum-treated cells immunostained for actin (2nd panel), cortactin (middle panel), and Src (4th panel). The arrowheads indicate active invadopodia initiation in the control cells and the cytoskeletal changes in the treated cells. Scale bars: 7.5 µm. (D and E) Western blotting analysis of TKS5, N-WASP, ARP3, ARP2, and CDC42, which are key proteins involved in the assembly stage of invadopodia formation, and MMP2 and MT1-MMP, which are involved in the maturation stage of invadopodia formation, after the cells were treated with Fucoidan-Sargassum (0, 10, 20, and 30 mg/mL) for 48 h.

Among the tested HCC cell lines, HCCLM3 is the most metastatic cell line; for this cell line, under a microscope, elongated protrusions known as invadopodia are noticeable (Figure 3A). Therefore, we chose HCCLM3 as the target cell line to use in the following study. According to invadopodia-related reviews, the maturation of invadopodia involves three stages of development, and each stage involves several key proteins 20. Src, focal adhesion kinase (FAK), and Cortactin are the key proteins in initiation stage. Src and FAK interact with integrins to establish focal adhesions with the ECM, and the aggregation of phosphorylated Cortactin by active Src at ECM sites of attachment are the crucial points of early invadopodia formation^21^, ^22^. We investigated the antineoplastic activity of Fucoidan-Sargassum and its effect on these key proteins by Western blotting and immunofluorescent staining. After treating the HCCLM3 cells with Fucoidan-Sargassum for 48 h, the expression levels of Src and Cortactin were downregulated, whereas the expression level of FAK was upregulated in a dose-dependent manner (Figure 3B). In the immunofluorescent analysis, the cells appeared morphologically distinct between the control and treated group. In the control group, the cells were stretched out in a triangular shape, the nuclei were large and round, and the targeted proteins Src and Cortactin were scattered and overlapped at the protrusion area. In contrast, in the treated group, the cell perimeters were smooth, round and diminished in size. The nuclei were deformed, the targeted proteins Src and Cortactin were decreased, and there was no trace of protrusions (Figure 3C).

In the assembly stage of invadopodia formation, Tks5 plays an important role in the anchoring step of the invadopodia precursor, and this protein may also serve as the scaffold that recruits cortactin to activate new actin polymerization, which involves actin regulatory proteins, including cofilin, Arp2/3, N-WASP, and Cdc42^23^. Thus, we selected Tks5, Arp2, Arp3, N-WASP, and Cdc42 as the key proteins to measure to further study the effect of Fucoidan-Sargassum on impeding the formation of invadopodia. The results revealed that the expression levels of all tested proteins exhibited the same pattern as that of Src and Cortactin, i.e., expression was downregulated in a dose-dependent manner, except for Arp2, which showed no change (Figure 3D).

In the maturation stage of invadopodia formation, recruitment of matrix metalloproteinases (MMPs) is the process that finalizes the formation of invadopodia^24^. Previous studies suggested that, in the event of invadopodia formation, Tks5 and cortactin act together to generate invasive protrusions and secrete metalloproteases. Subsequently, Tks4 stabilizes and localizes MT1-MMP to activate the functions of the MMPs at the focal site, leading to the proteolytic degradation of the ECM 25. To investigate the inhibitory effect of Fucoidan-Sargassum on invadopodia formation, we analyzed the expression of MMPs proteins. The results revealed that the expression levels of MT1-MMP and MMP2 were downregulated in the same pattern as we observed earlier for Tks5 and Cortactin (Figure 3E).

### Fucoidan-Sargassum downregulates the Src signaling pathway by inactivating integrin αVβ3

**Figure 4:**
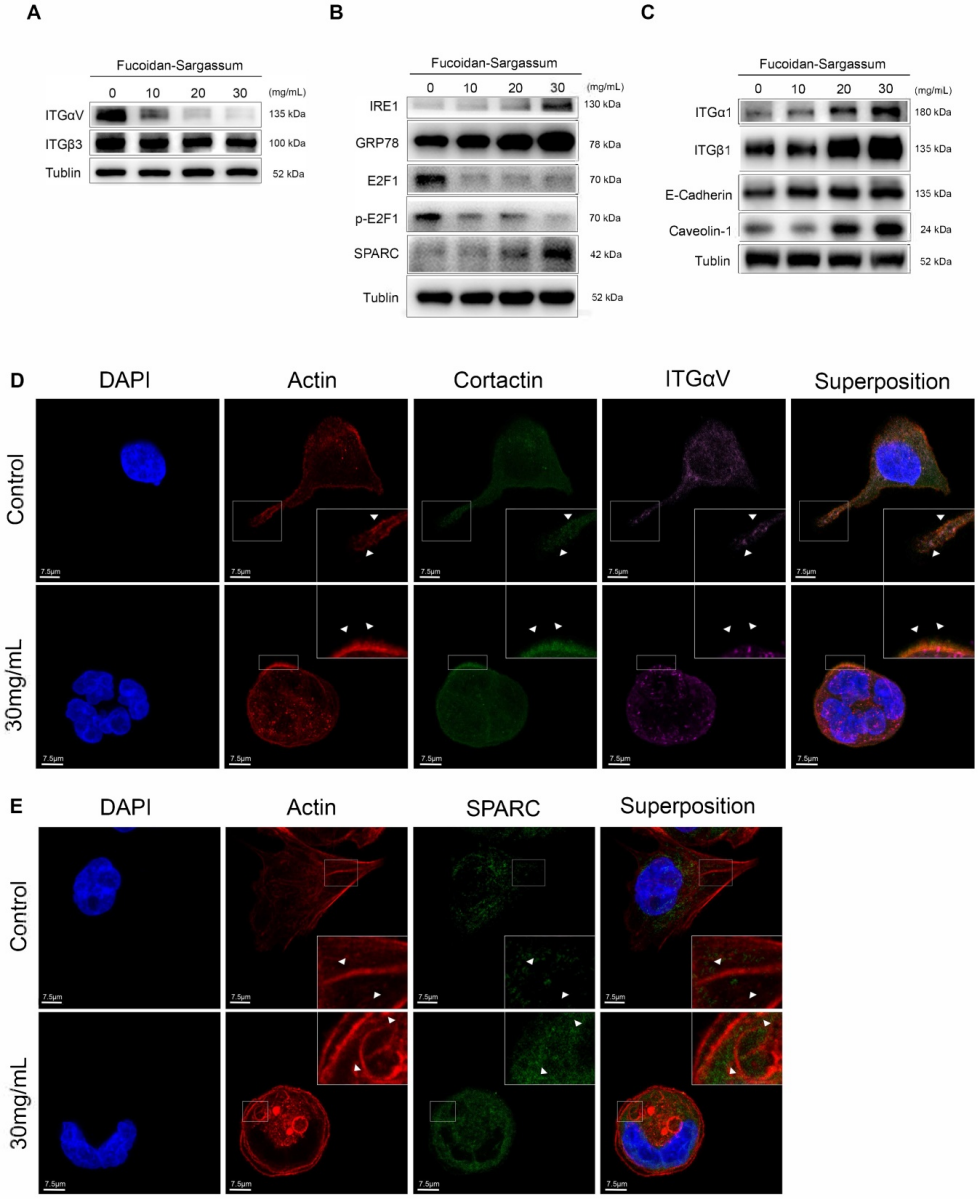

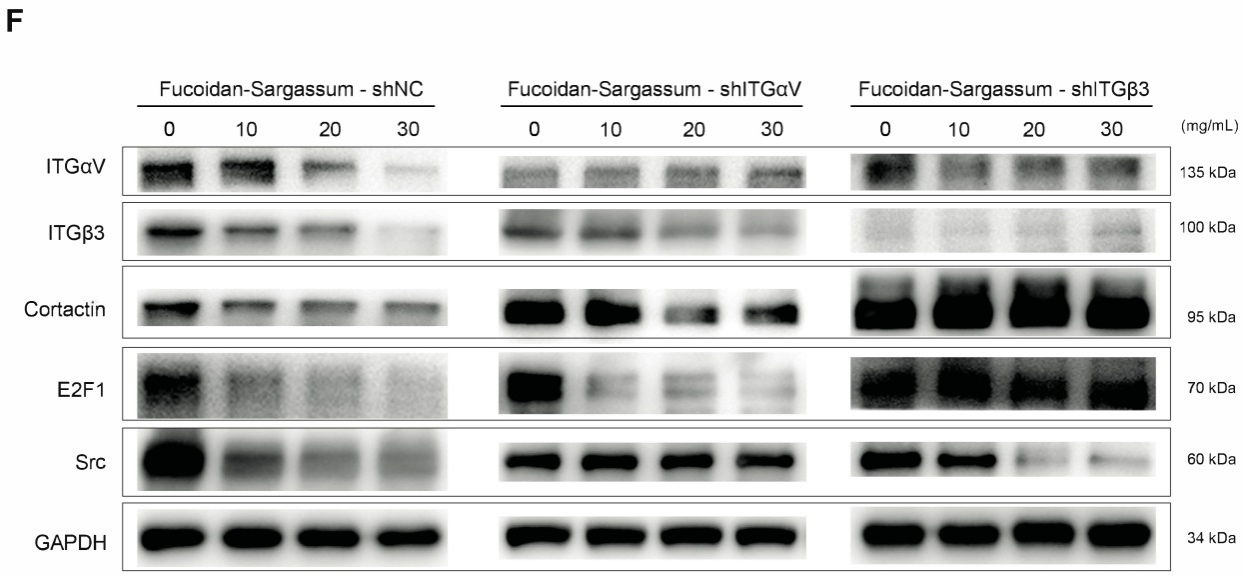
In HCCLM3 cells, Fucoidan-Sargassum inhibits the maturation stages of invadopodia formation, deactivates integrin αVβ3 and transcription factor E2F1, induces endoplasmic reticulum stress, and enhances cell-ECM adhesion. (A-C) Western blotting analysis of the integrin receptors αVβ3, the transcription factor E2F1, the key proteins of endoplasmic reticulum stress GRP78, IRE1, and SPARC, and ECM collagen-related integrin α1β1 and cell adhesion proteins E-Cadherin and Caveolin-1, after the cells were treated with Fucoidan-Sargassum (0, 10, 20, 30 mg/mL) for 48 h. (D) Confocal images of fixed control cells or Fucoidan-Sargassum-treated cells immunostained for actin (2nd panel), cortactin (middle panel), and integrin αV (4th panel). The arrowheads indicate active invadopodia in the control cells and cytoskeletal changes in the treated cells. (E) Confocal images of fixed control cells or Fucoidan-Sargassum-treated cells immunostained for actin (2nd panel), and SPARC (3rd panel). Note that the localization of SPARC in the control and treated cells is identified by arrowheads. Scale bars: 7.5 µm. (F) Western blotting analysis of ITGαVβ3/Src/E2F1 signaling pathway after the cells were transfected with shITGαV and shITGβ3, and treated with Fucoidan-Sargassum (0, 10, 20, 30 mg/mL) for 48 h.

After confirming that Fucoidan-Sargassum impedes invadopodia formation through mediating Src kinase and its downstream proteins, we next asked which upstream receptors were involved. To answer this question, we searched through previous studies, which revealed that the integrin adhesion receptors and Src family protein tyrosine kinases (SFKs) act together to transmit extracellular signals into the cells. In particular, SFKs play a key role in initiating such signaling by interacting directly with integrins without binding to FAK 26. Therefore, we examined several α and β integrins, and we found that the expression levels of αV and β3 followed the same pattern as those of Src in our earlier findings (Figure 4A). In the immunofluorescence analysis, similar to our findings regarding Src and Cortactin, the cells appeared morphologically distinct between the control and treated group. In the control group, the cells exhibited an obvious elongated protrusion, the nuclei were round, and the targeted proteins ITGαV and Cortactin were scattered and overlapped at the protrusion area. In contrast, in the treated group, the cell surfaces were smooth, round, and diminished in size. The nuclei were deformed and clustered, the targeted proteins ITGαV and Cortactin were scattered in places, and there was no trace of protrusions (Figure 4D).

### Fucoidan-Sargassum suppresses transcription factor E2F1 and induces Endoplasmic Reticulum (ER) stress

In the previously mentioned immunofluorescence experiment, we observed deformed nuclei in the treated group; thus, we hypothesized that in addition to impeding invadopodia formation, Fucoidan-Sargassum likely impacts the HCCLM3 cell line at the level of the nucleus. We analyzed the expression of several transcription factors, and our findings revealed that the expression level of E2F1 was markedly downregulated (Figure 4B). Next, we measured the expression of GRP78/BIP, IRE1, and SPARC to further investigate the impact of Fucoidan-Sargassum within cells. We selected these proteins because many studies have revealed that E2F1 and glucose-regulated protein 78 (GRP78/BIP) have an inverse relationship, wherein E2F1 promotes apoptosis and GRP78/BIP plays a key role in cell survival 27, 28. Moreover, during ER stress, GRP78/BIP, which is a master regulator of the unfolded protein response (UPR), becomes active after dissociating from IRE1, PERK, and ATF6 (the three sensors of the UPR). Among these three sensors, only IRE1 has a role in impacting cell migration and invasion through IRE1-dependent decay (RIDD)^29^. Recently, various RIDD targets have been discovered in pathways that promote tumor growth and metastasis; basement membrane protein 40 (BM-40), also known as secreted protein acidic and rich in cysteine (SPARC), is one such target 30. SPARC orchestrates a wide range of cellular functions, such as proliferation, survival, migration, and adhesion. SPARC also regulates integrin (α, β) clustering and activation, ECM assembly, growth factor signaling, and the cross-talk between integrins, ECM, and growth factor receptors (RTK) 31. Our results indicated that the expression levels of all target proteins were upregulated in a dose-depend manner (Figure 4B). In the immunofluorescence analysis, once again the cells appeared morphologically distinct between the control and the treated group. In the control group, the cells appeared as fighter jet-like with sharp edges, the nuclei were large and round, and only a few spots that were positive for the target protein SPARC appeared close to the protrusion area. In contrast, in the treated group, the cell borders were smooth, round and diminished in size. The nuclei were deformed, and the abundance of SPARC was twice as high compared with the control group (Figure 4E).

### Fucoidan-Sargassum activates integrins α1β1 and enhances cells adhesion

Though immunofluorescence analysis, we observed that the cells were originally either triangular or spindle shaped before the treatment with Fucoidan-Sargassum. After the treatment, the shape of the cells changed to round or oval. These morphological changes may indicate that Fucoidan- Sargassum promotes the cytoskeletal change from EMT to MET. Therefore, we further examined other integrins that may also participate in this transformation. We found that the levels of integrin α1β1 were elevated in a dose-dependent manner (Figure 4C), similar to the findings we observed earlier for FAK (Figure 3B). FAK and integrins play a role in cell-cell and cell-ECM adhesion, and E-cadherin and caveolae also regulate the migratory capability of cells. The expression levels of E-cadherin and Caveolin-1 were also shown to be elevated in a dose-dependent manner, similar to integrins α1β1 (Figure 4C).

### Integrins αVβ3 are the direct targets of Fucoidan-Sargassum and mediate the Src/Cortactin/E2F1 signaling pathway

To verify whether the integrins αVβ3 are direct targets of Fucoidan-Sargassum, lentivirus-mediated ITGαV-shRNA and ITGβ3-shRNA were transfected into HCCLM3 cells, and the protein expression levels of Src, Cortactin, E2F1 were evaluated. Interestingly, after the HCCLM3 cells were transfected with ITGαV-shRNA, the expression levels of Src did not decrease in a dose dependent manner, in contrast to the control cells. However, the levels of Cortactin and E2F1 decreased in a dose dependent manner. In contrast, in the cells that were transfected with ITGβ3-shRNA, the expression levels of Src did decrease in a dose dependent manner, which was similar to the control cells; however, the expression levels of Cortactin and E2F1 exhibited no changes. Taken together, these findings indicate that ITGαV regulated Src but had no effect on Cortactin or the transcription factor E2F1. In contrast, ITGβ3 had no effect on Src but did affect Cortactin and E2F1; therefore, we conclude that integrins αVβ3 must bind to facilitate invadopodia formation, as many studies have reported that integrins αVβ3 were found activated in malignant cancer cell lines (Figure 4F).

### Fucoidan-Sargassum inhibits metastatic lung cancer *in vivo*

**Figure 5:**
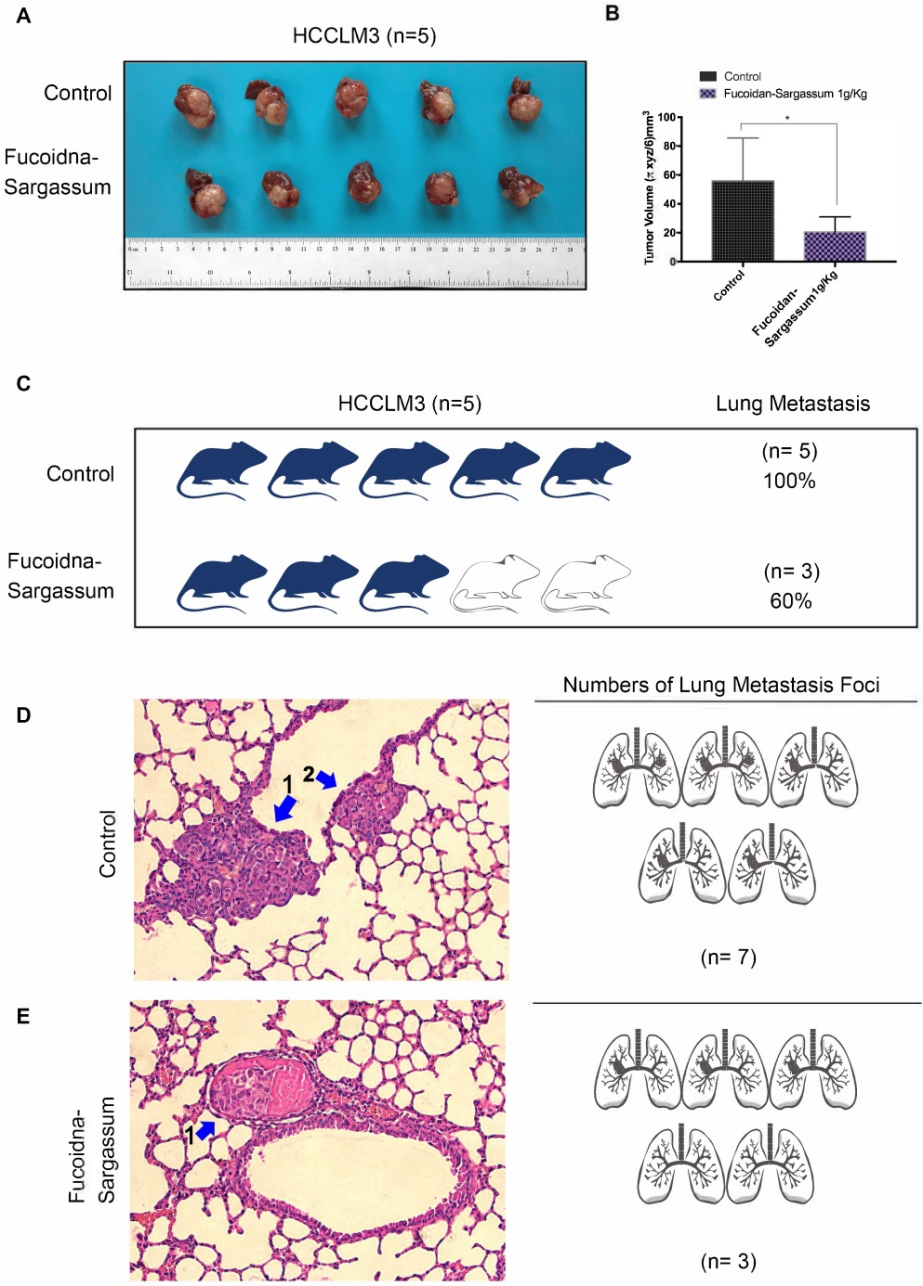
Fucoidan-Sargassum reduces the number of lung metastases in nude mice. (A) A total of 10 liver tumors, 5 tumors each from the control and the treated groups. A hepatocellular carcinoma xenograft model was used in 5-week-old nude mice. Mice were given Fucoidan-Sargassum (1 g/kg) by oral administration and were sacrificed on the 25th day. (B) The tumor volumes were calculated using the following formula: Tumor volume = π xyz/6 mm^3^. The data are expressed in a bar graph ((*P < .05 vs. control by t-test). (C) The image represents the number and the percentage of mice that have lung tumor metastasis. (D and E) Lungs were subjected to histological analysis (H&E stain) for the quantification of lung metastatic foci.

A hepatocellular carcinoma xenograft model was used to explore the effect of Fucoidan-Sargassum on tumor growth and metastasis *in vivo*. After five weeks of oral administration of Fucoidan-Sargassum, the liver tumor tissues were smaller in the Fucoidan- Sargassum group compared with the control group (volume, 55.8 ± 13.32 vs. 20.6 ± 4.675 mm^3^, P < 0.05, Figure 5A and 5B). Furthermore, we questioned whether there was a difference in the occurrence of metastatic lung cancer between the control and the treated groups; thus, histopathological examinations of lung samples were performed. The examinations revealed that the mice in the treated group had less frequent occurrence of metastatic lung cancer (3 of 5, 60%) compared to the control group (5 of 5, 100%) (Figure 5C). In addition, the lung metastatic foci were counted in both groups, and 2.3-fold fewer foci were found in the treated group compared with the control group (treated 3 vs. control 7, Figure 5D). Taken together, these results suggest that Fucoidan- Sargassum suppresses HCC tumor growth and lung metastasis in the host's body.

## Discussion

This study demonstrated that Fucoidan-Sargassum inhibits metastatic behavior in the HCC cell lines Huh7, SMMC-7721, and HCCLM3 (Figure 2A-2E). The mechanism underlying the Fucoidan-Sargassum-induced inhibition of invadopodia formation in HCCLM3 cells was determined to involve the deactivation of the integrin αVβ3/SRC/E2F1 signaling pathway. Furthermore, Fucoidan-Sargassum treatment led to a reduced number of lung metastatic foci in a HCCLM3 hepatocellular carcinoma xenograft model (Figure 5C -5E).

**Figure 6:**
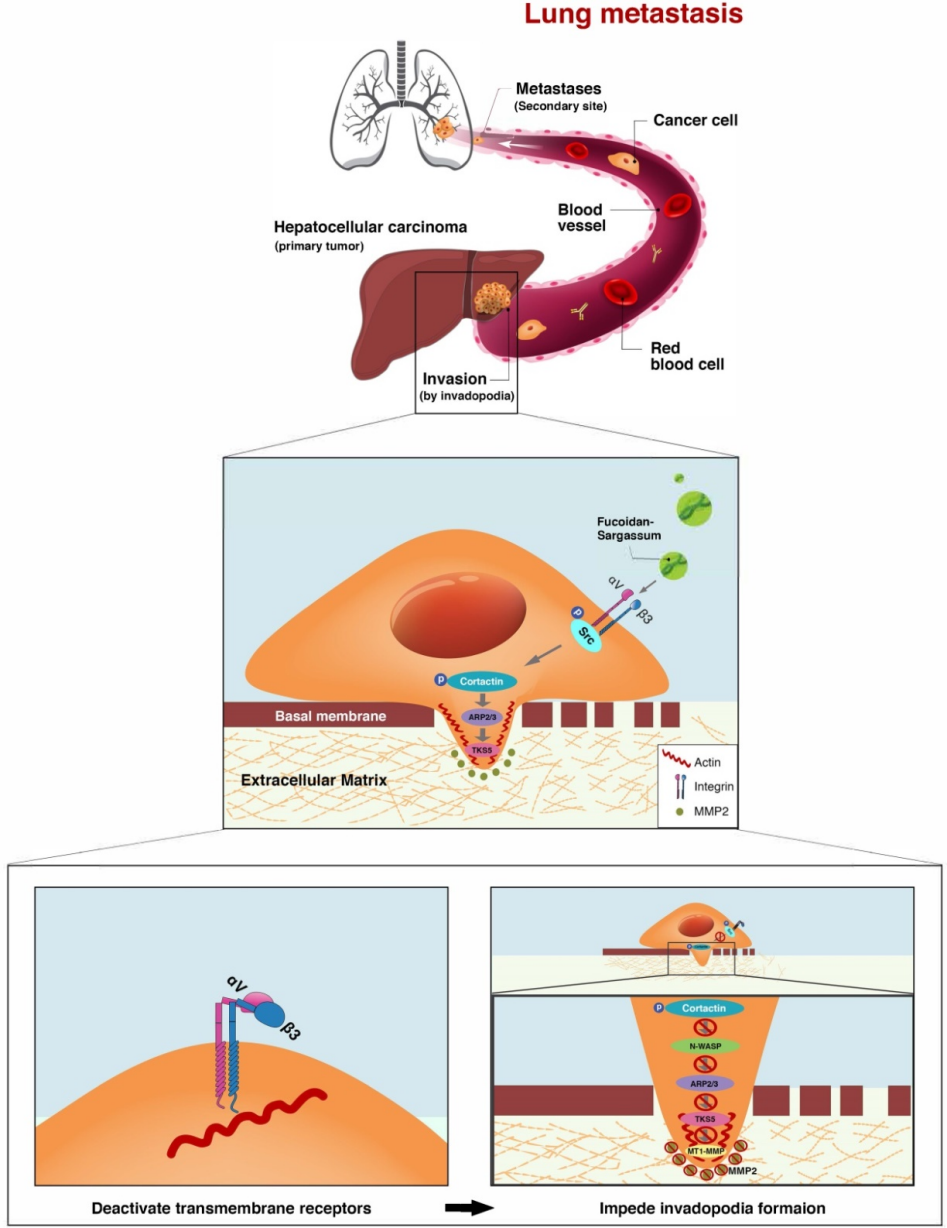
The proposed mechanism through which Fucoidan-Sargassum impedes invadopodia formation in HCCLM3 cells. Fucoidan-Sargassum deactivates integrin αVβ3, thereby disabling the Src kinase signaling pathway and downregulating key proteins related to invadopodia formation.

Integrin receptors are situated between the cytoplasm and the extracellular space and directly facilitate cell-cell and cell-ECM adhesion. When the integrin α and β subunits bind tougher, cellular signal transduction pathways are activated 32. Recent studies have reported that tumor tissues which express integrin αVβ3 tend to metastasize aggressively, and this complex has been detected in several cancers, including in HCC 33, 34. Our results show that Fucoidan-Sargassum impedes invadopodia formation by deactivating integrin αVβ3 (Figure 4A), thereby disrupting cell signaling through the integrin-dependent Src kinase pathway, transducing the signal from outside of the cell into the cell (Figure 6).

**Figure 7:**
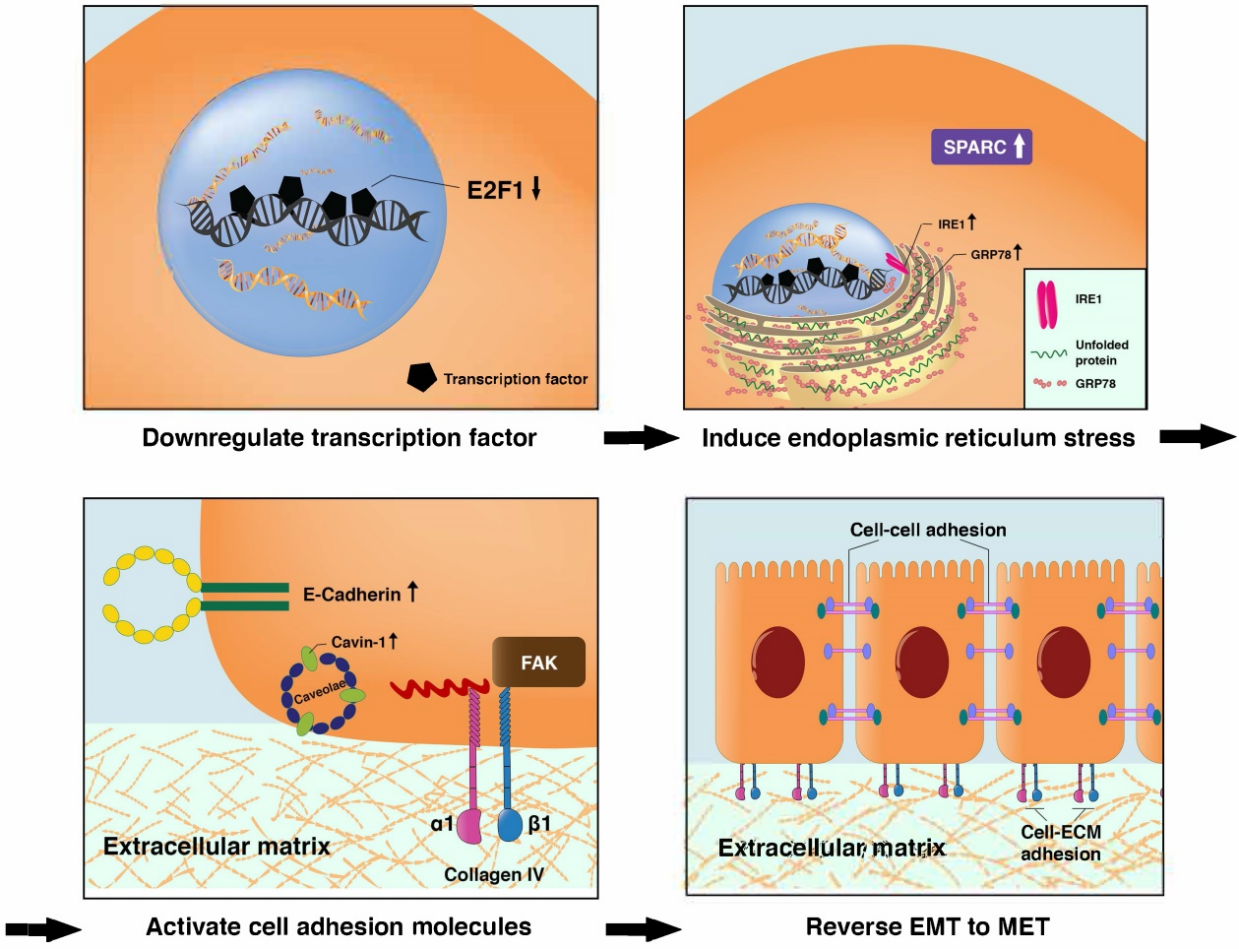
Proposed transitional events that occurred in HCCLM3 cells after treatment with Fucoidan-Sargassum, including the induction of endoplasmic reticulum stress, the activation of ITGα1β1 and the enhancement of cell-cell and cell-ECM adhesion.

It is reasonable, if not logical, to assume that the antimetastatic effects of Fucoidan-Sargassum are exerted through the following pathway, ITGαVβ3/ SRC/ E2F1/ GRP78/ IRE1/ SPARC/ ITGα1β1, from deactivating integrin αVβ3 to activating the integrin α1β1 signaling pathways (Figure 6 and 7). E2F1 is well-known for its role in regulating cell-cycle progression and inducing apoptosis in response to DNA damage 35. Recent research uncovered an additional function of E2F1; under the influence of certain cofactors, E2F1 activates the growth receptor signaling pathways, resulting in cancer progression and chemo resistance 36. Multiple studies have shown that overexpressed E2F1 is associated with high-grade tumors or metastasis and is observed in many types of human cancer, such as breast, lung, and HCC. Our findings indicate that Fucoidan- Sargassum reduced the expression level of E2F1 in the HCCLM3 cell line (Figure 4B); therefore, Fucoidan- Sargassum may have inhibited tumor progression by leading the cancer cells to arrest (Supplementary figure 1A). Since the endoplasmic reticulum (ER) is prominent in hepatocytes, we investigated ER stress-related proteins. Strikingly, we found that HCCLM3 cells were under ER stress after treatment with Fucoidan- Sargassum. Many studies have proposed using ER stress as a therapeutic target; in particular, it may be more effective to use drug combinations that maximize ER stress and proteotoxicity 37. Another intriguing finding in the present study was that SPARC/BM-40 was upregulated after ER stress was induced. Recent studies showed that, in liver cancer, overexpression of SPARC/BM-40 in HCC cells induced MET and resulted in reduced tumorigenesis 38. Therefore, we speculate that SPARC was the protein that initiated the activation of integrin α1β1 (a type IV collagen receptor) signals. Surprisingly, a recent review proposed that SPARC/BM-40 functions as an ECM chaperon-like protein to ensure that collagen IV is properly deposited and polymerized into nascent laminin networks and to prevent collagen IV from assembling prematurely at the sites of production 39. This new knowledge helped us to interpret the results obtained in our study.

An interesting finding worth mentioning is that Fucoidan- Sargassum may activate the adhesive crosstalk between Integrin α1β1 and E-cadherin, as the expression levels of both proteins were shown to be upregulated in this study (Figure 4C). Previous studies revealed that the interactions between cadherins and integrins (two distinct types of adhesion) constructed cell-cell and cell-ECM adhesive networks to alter cellular or physiological condition within the cell, such as proliferation, collective cell migration, epithelial- mesenchymal transition, and many others^40^. Moreover, the increased expression of ECM leads to integrin engagement, the activation of FAK and the subsequent promotion of E-cadherin expression 41. Therefore, this may be the reason why FAK expression was upregulated, as integrin α1β1 and E-cadherin presented in our evidence (Figure 3B and 4C). Like FAK, caveolae has more than one function within cells, including the regulation of cell growth, cell migration, endocytosis and lipid trafficking. Many studies have demonstrated that caveolin-1 overexpression is associated with HCC tumorigenesis and metastasis and results in the downregulation of E-cadherin and the upregulation of MMP2 and MT1-MMP 42. In our study, caveolin-1 was the only protein for which the expression pattern did not coincide with other HCC studies. Instead, caveolin-1 was overexpressed in a dose-dependent manner after treatment with Fucoidan-Sargassum. The reason for this contradictory result may be because caveolin-1 is synthesized as an integral membrane protein and oligomerized in the endoplasmic reticulum, and its functional role is linked to its trafficking and dynamics 43. Earlier, we mentioned that Fucoidan-Sargassum induced endoplasmic reticulum stress, and this could be the factor that alters the functional role of caveolin-1; this topic warrants further study.

Last but not least, we felt it was important to investigate the therapeutic effects of Fucoidan- Sargassum on pro-metastatic target genes and proteins that were previously reported in clinical liver cancer studies. For example, MPZL1/PZR was identified as the target gene that plays a pivotal role in HCC tumor metastasis 44; GP96 was reported as an oncogenic chaperone in hepatocytes that promotes hepatocellular carcinogenesis 45; and KLF-4 was demonstrated to be a promising target to treat cancers, including HCC 46. In addition to testing therapeutic targets, we were also curious about the supportive effect of Fucoidan-Sargassum; thus, we investigated the expression of HDAC6, which has been shown to be suppressed or lost in HCC, which influences the balance of autophagic signals. Thus, activating HDAC6 could potentially lead to autophagic cell death during hepatocarcinogenesis 47. We were pleased to find that Fucoidan- Sargassum is not only antineoplastic regarding the aforementioned targeted genes and proteins, but it also activated HDAC6 signaling (supplementary figure 1B and 1C).

In conclusion, this study demonstrates that Fucoidan-Sargassum exhibits no cytotoxicity in normal liver cell, it impedes invadopodia formation by deactivating the integrin αVβ3/ SRC/ E2F1 signaling pathway and enhances cells adhesion by activating integrin α1β1 and E-cadherin signals in the HCCLM3 cell line. Further *in vivo* and clinical studies are warranted for the development of a novel antineoplastic agent or a cocktail therapy for treating liver cancer, thereby providing more options for preventing cancer metastasis and recurrence. Taken together, our findings indicate that Fucoidan- Sargassum may be a promising therapeutic antimetastatic compound in the development of a metastasis-preventive drug for liver cancer.

## Supplementary Material

Supplementary figure.

## Figures and Tables

**Table 1:** Inhibitory effect of Fucoidan-Sargassum on HCC and normal liver cell lines

		Inhibition rate of Fucoidan-Sargassum in dose increments			
Time	Cell line	5mg/mL	10mg/mL	20mg/mL	30mg/mL
h		%	%	%	%
24	HL-7702†	2.8	2.4	2.1	___
	Huh7	0.3‡	9.3*	23.2***	___
	SMMC-7721	___ ‡	6.4	17.6**	28.4***
	HCCLM3	___	12.9*	24.1***	24.9***
					
48	HL-7702†	2.8	4.1	6.9*	___
	Huh7	7.3*	21.7***	40.2****	___
	SMMC-7721	___	8.2	17.8**	28.2***
	HCCLM3	___	13.4**	27.9***	43.8****
					
72	HL-7702†	3.1	4.8	6.4*	___
	Huh7	4.5	32.2***	48.8****	___
	SMMC-7721	___	7.8*	29.2***	46.9****
	HCCLM3	___	12.1**	35.7****	51.3****

Data are inhibition rates of 3 dose increments for 4 cell lines. †Normal liver cell line. ‡Not determined. *P < .05, ** P < .01, *** P < .001, **** P < .0001 vs. control, by two-way ANOVA.

## Abbreviations

ITG: integrin
BM-40: basement membrane protein 40
EMT: epithelial-mesenchymal transition
ER: endoplasmic reticulum
FAK: focal adhesion kinase
GRP78: glucose-regulated protein 78
HCC: hepatocellular carcinoma
MET: mesenchymal- epithelial transition
RIDD: IRE1-dependent decay
SPARC: secreted protein acidic and rich in cysteine
