# Supplementary material for: Antimetastatic Effect of Fucoidan-Sargassum against Liver Cancer Cell Invadopodia Formation via Targeting Integrin αVβ3 and Mediating αVβ3/Src/E2F1 Signaling
Source: J Cancer. 2019 Aug 27;10(20):4777–92. doi: 10.7150/jca.26740 (PMC6775528; doi:10.7150/jca.26740)

Supplementary figure 1. The potential of Fucoidan-Sargassum to be integrated into hepatocellular carcinoma treatment regimens. (A) The images show that Fucoidan-Sargassum markedly increased apoptotic bodies in a dose-dependent manner, compared to the control cells. (B) Fucoidan-Sargassum inhibited pro-metastatic target genes and proteins. (C) Fucoidan-Sargassum activated HDAC6, which is related to autophagic cell death during hepatocarcinogenesis.

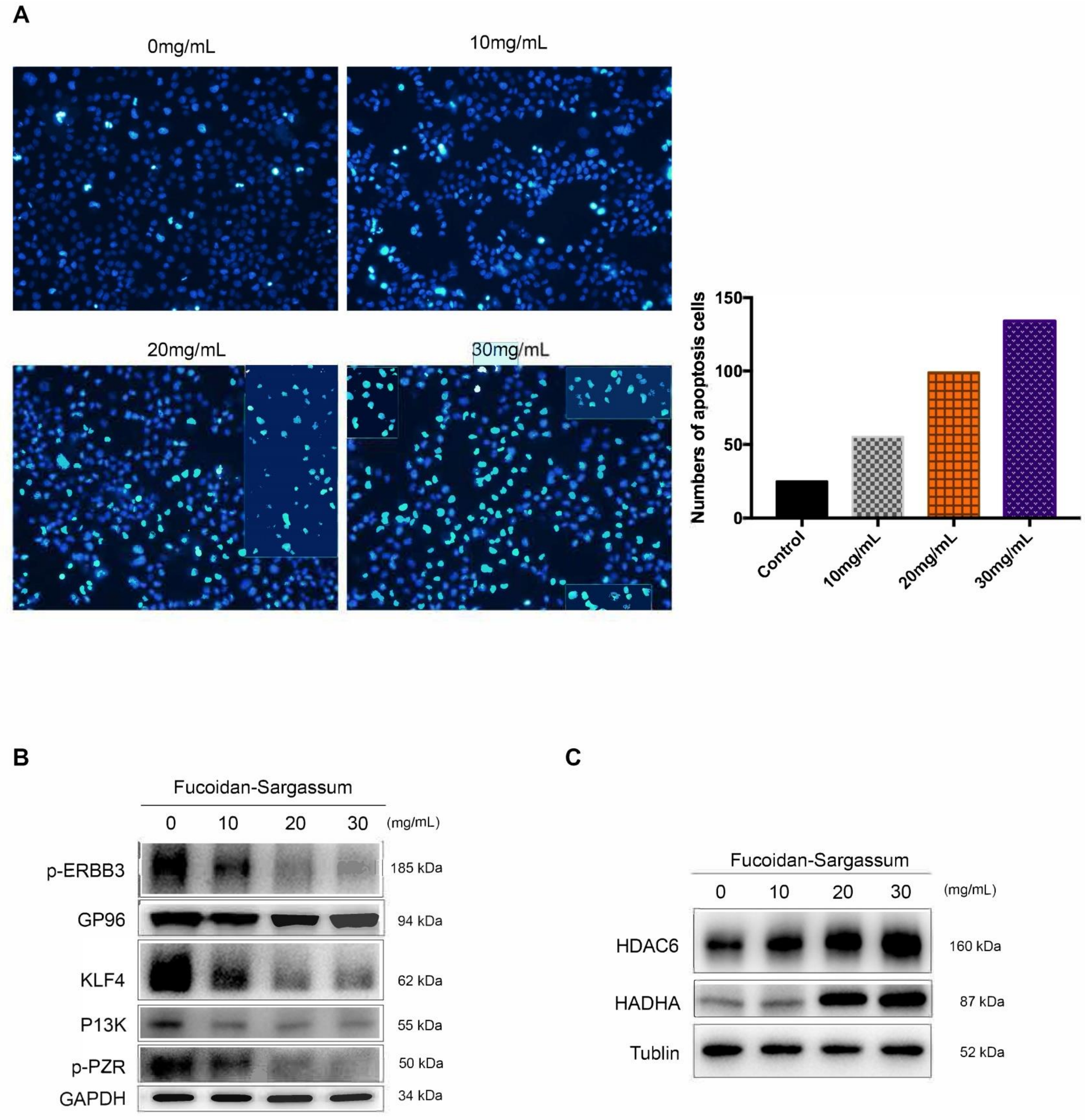

Supplement: Supplementary file 1 — Supplementary figure. [file jcav10p4777s1.pdf]
